# Social Isolation, Caregiving Alone, and Caregiving Stress in Family Caregivers of Older Adults in Korea

**DOI:** 10.1093/geroni/igab046.3525

**Published:** 2021-12-17

**Authors:** Sol Baik, Jiweon Jun

**Affiliations:** 1 University of Maryland, Baltimore, Baltimore, Maryland, United States; 2 Seoul National University, Center for Transnational Migration and Social Inclusion, Seoul National University, Seoul-t'ukpyolsi, Republic of Korea

## Abstract

The tendency of caregiving alone is increasing, and these solo caregivers often perceive caregiving responsibilities as a burden. Still, literature on positive aspects of caregiving shows that not all caregivers experience severe distress. Little is known on which factors make a difference in experiencing caregiving distress among solo caregivers. We focused on the empirical findings on the negative impact of social isolation on caregiver’s mental health, examining if and how the intersection of solo caregiving and social isolation is related to severe caregiving stress among caregivers of older adults in Korea. We analyzed 501 family caregivers of older adults in Korea using survey data from the Care Work and the Economy research project (2018). We conducted ordinal logistic regression analysis. The findings show that solo caregivers with a lack of social time fall under the most at-risk group of caregivers in terms of experiencing severe stress (OR=3.72, SE=0.93) whereas solo caregivers with enough social time did not show significantly higher stress compared to the reference group (OR=1.50, SE=0.43). Being socially isolated caregivers still had high levels of stress despite the division of care (OR=2.16, SE=0.55), implying the need to provide caregivers more time for social interaction with others. The current public long-term care insurance in Korea provides limited hours of in-home care aide services to enable aging in place of older adults. To reduce the social isolation of caregivers, it is necessary to extend the service hours and provide support, such as creating online caregiver networks.

